# Space-Wave Routing via Surface Waves Using a Metasurface System

**DOI:** 10.1038/s41598-018-25967-8

**Published:** 2018-05-15

**Authors:** Karim Achouri, Christophe Caloz

**Affiliations:** Ecole polytechnique de Montreal, Department of Electrical Engineering, Montreal, QC H3T 1J4 Canada

## Abstract

We introduce the concept of a metasurface system able to route space waves via surface waves. This concept may be used to laterally shift or modulate the beam width of scattered waves. The system is synthesized based on a momentum transfer approach using phase-gradient metasurfaces. The concept is experimentally verified in an “electromagnetic periscope”. Additionally, we propose two other potential applications namely a beam expander and a multi-wave refractor.

## Introduction

Metasurfaces are thin electromagnetic films composed of flat scatterers and represent the two-dimensional counterparts of volume metamaterials^1,2^. Over recent years, they have attracted tremendous attention due to their unprecedented capabilities to control electromagnetic waves conjugated with their ease of fabrication, low loss and high compactness.

The vast majority of metasurface designs and applications reported to date have been restricted to isolated metasurfaces, i.e. single metasurface structures performing specific electromagnetic transformations. In order to extend the range of these transformations, we propose here the concept of a *metasurface system*, namely a combination of several metasurface structures collectively exhibiting properties that would be unattainable with a single metasurface. Specifically, we present a metasurface system composed of three juxtaposed metasurfaces, that *routes* space-wave beams, between different locations, via surface waves^3,4^. Such a system may be used, for instance, to laterally shift or modulate the beam width of scattered waves. The proposed space wave routing concept was theoretically introduced in^5^ using the synthesis technique reported in^6^.

This paper is organized as follows. We start by introducing the concept of space-wave routing via surface waves in a metasurface system. Next, we present the synthesis technique used for the design of such a system. We then experimentally demonstrate system routing in an “electromagnetic periscope” and present the corresponding design and measurements. Finally, we discuss two additional potential applications, namely a compact beam expander and a multi-wave refractor.

## Results

### Space-Wave Routing Concept

**Figure 1:**
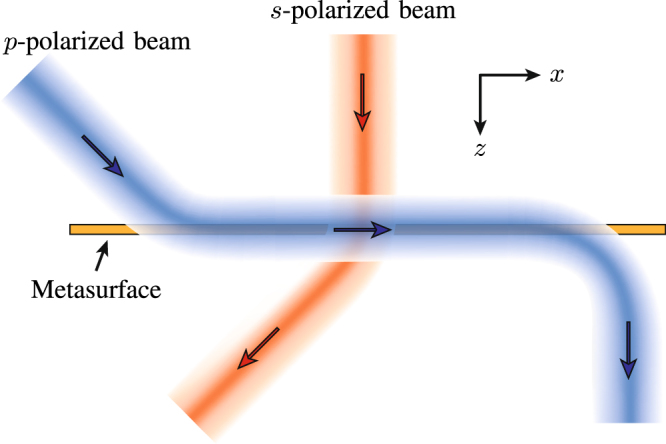
Concept of metasurface system performing the operations of space-wave routing via surface waves for *p*-polarized beams and generalized refraction for *s*-polarized beams.

The fundamental idea, which is depicted in Fig. 1, consists in converting an incoming space wave into a surface wave, propagating this surface wave between two points along a desired path, and then converting it back, with possible other transformations, into an outgoing space wave. This concept may be used to laterally shift reflected or transmitted waves (electromagnetic periscope), modulate the width of beams, or enable multiple refraction, in a very compact fashions, as will be discussed thereafter.

In the system depicted in Fig. 1, the metasurface is assumed to be monoanisotropic diagonal, and hence birefringent, allowing for the independent control of *s* and *p* polarizations. The metasurface may be designed, for instance, to route *p*-polarized waves and refract (or perform any another transformation on) *s*-polarized waves.

**Figure 2:**
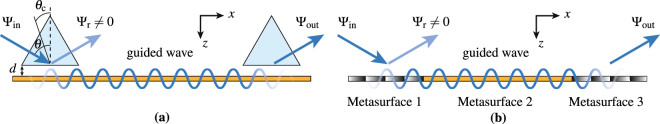
Representations of two optical systems performing the same wave routing operation. (**a**) Combination of two prisms and a dielectric waveguide. (**b**) Composite metasurface, including two spatially modulated metasurfaces placed at the ends of a guiding metasurface.

We shall now describe the space-wave routing concept in more details. Let us consider the optical system depicted in Fig. 2a, which consists of a dielectric waveguide with two prisms placed at different locations above it. This system may be used to perform the routing operation described in Fig. 1. Assume that an input beam Ψ_in_ is impinging on the left prism at an angle *θ* > *θ*_c_, where *θ*_c_ is the angle of total internal reflection. An evanescent wave with wavenumber *k*_*x*_, corresponding to that of the incident wave, is formed between the prism and the waveguide due to total internal reflection. This evanescent wave then couples to a waveguide mode with matched *k*_*x*_, and the resulting wave propagates along the waveguide in the +*x*-direction. The amount of coupling between the incident space wave and the guided wave is proportional to the distance *d* between the prism and the waveguide, and is usually less than unity, leading to a non-zero reflected wave Ψ_r_. Farther along the waveguide, the guided wave is transformed back into an output space wave Ψ_out_ by the second prism by the reverse mechanism.

We introduce here the metasurface system depicted in Fig. 2b to perform the same operation in a much more compact (purely planar) and (ideally) perfectly reflection-less fashion. This system consists of three different metasurfaces juxtaposed to each other. The first metasurface, on the left in the figure, is a spatially modulated structure with periodicity in the *x*-direction. When the incident wave, Ψ_in_, illuminates this metasurface, the latter generates an evanescent wave with a propagation constant in the *x*-direction larger than the free-space wavenumber (slow wave). Ideally, this metasurface should pick up the incident space wave, *without re-radiation*, in contrast to the prism in Fig. 2a, since the corresponding re-radiation/reflection represents routing loss. The second metasurface in the figure is a waveguiding structure designed with *x*-wavenumber equal to that of the first structure so that the evanescent wave generated by the first metasurface couples into. The second metasurface routes then the wave towards the third metasurface. Finally, the spatially modulated third metasurface transforms the guided wave back into a space wave at the other end of the system through the discontinuity.

Note that it is also possible to implement the space-wave to surface-wave coupling mechanism in Fig. 2a by replacing the prisms with phase-gradient metasurfaces that would lie on top of the waveguiding structure instead of being adjacent to it as in Fig. 2b. Such a design has been demonstrated in^7^ and leads to a space wave to surface-wave conversion efficiency greater than that of Fig. 2b at the cost of a bulkier system.

### Metasurface System Synthesis Approach

In this section, we succinctly describe the synthesis approaches that we have used to realize the metasurface system. There are two different types of metasurfaces composing that system: a waveguiding structure (second metasurface in Fig. 2b), and two space-wave to surface-wave metasurface converters (first and third metasurfaces in Fig. 2b).

The waveguiding structure is relatively simple to implement. In order to realize it, we have followed the well known design procedures routinely used in the implementation of slow-wave structures^8,9^. The general idea consists in numerically optimizing the metasurface scattering particles, using an eigenmode solver, such that it supports the propagation of a surface wave with the desired frequency, propagation constant and polarization. The main difference, compared to conventional techniques, is that we wish to achieve waveguiding for one specific polarization, while for the other polarization, the metasurface must be transparent. This means that the metasurface must be anisotropic, which complicates the optimization procedure.

**Figure 3:**
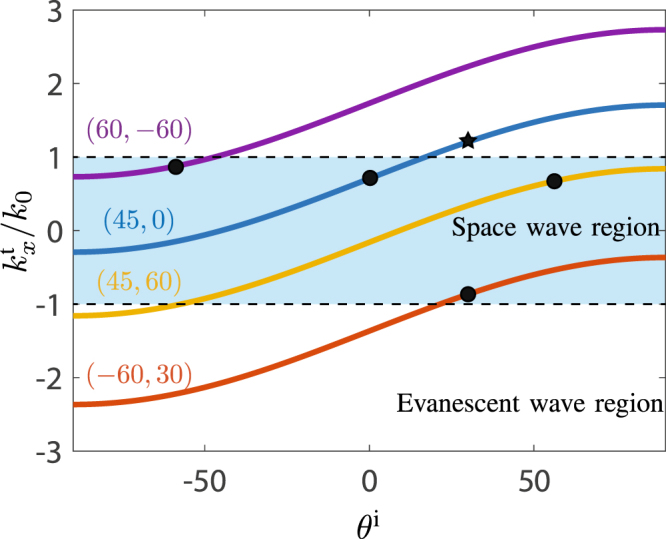
Normalized transverse wavenumber of the transmitted wave versus incidence angle in the phase-gradient metasurface for several specified angles (*θ*^t,spec^, *θ*^i,spec^) with specification indicated by a black circle.

The space-wave to surface-wave metasurface converters are realized using almost the same scattering particles as those of the waveguiding metasurface. The only difference is that the shape of these particles are modulated so as to achieve coupling between space waves and surface waves so that the metasurface converters act as leaky-wave antennas. The spatial modulation of the scattering particles takes here the form of a simple phase gradient^10^. To understand how the coupling occurs, let us consider the generalized law of refraction^11^. It can be expressed, using the transverse wavenumber of the incident and refracted waves and the effective wavenumber of the phase-gradient structure *K*, as

1 $${k}_{x}^{{\rm{t}}}={k}_{x}^{{\rm{i}}}+K,$$

where *K* = 2*π*/*P* with *P* being the phase-gradient period of the metasurface. This period is designed such that the specified incident wave is refracted at a specified angle, i.e. *P* = *λ*_0_/(sin*θ* ^t,spec^−sin*θ* ^i,spec^). From (1), we express the normalized transverse wavenumber of the transmitted wave as a function of *K* and the incidence angle as

2 $$\frac{{k}_{x}^{{\rm{t}}}}{{k}_{0}}=\,\sin \,{\theta }^{{\rm{i}}}+\frac{K}{{k}_{0}}=\,\sin \,{\theta }^{{\rm{i}}}+\,\sin \,{\theta }^{{\rm{t}},\mathrm{spec}}-\,\sin \,{\theta }^{{\rm{i}},\mathrm{spec}},$$

which allows to determine the transverse wavenumber for *any* incidence angle *θ*^i^. As an illustration, relation (2) is plotted in Fig. 3 as a function of the incidence angle and for different specified angle pairs.

The region in blue, where $|{k}_{x}^{{\rm{t}}}/{k}_{0}| < 1$, corresponds to space-wave modes. Outside of this region, $|{k}_{x}^{{\rm{t}}}/{k}_{0}|$ is larger than 1 and the longitudinal wavenumber ${k}_{z}^{{\rm{t}}}=\sqrt{{k}_{0}^{2}-{({k}_{x}^{{\rm{t}}})}^{2}}$ is therefore imaginary, corresponding to a *z*-evanescent or surface-wave mode. This shows that a simple phase-gradient metasurface can be used as a converter between a space wave and a surface wave when the metasurface wavenumber *K* and the incidence angle *θ*^i^ are properly chosen^3,4,12^. Note that the concept of using phase-gradient metasurfaces to generate surface wave from space waves also holds for acoustic waves, as discussed in^13^.

The three-metasuface system in Fig. 2b may therefore be realized as follows. The first metasurface is designed as a phase-gradient metasurface with increasing phase in the +*x*-direction; this positive phase ramp increases the momentum of the incident wave (in the *x*-direction) so as to transform it into a surface wave. The second metasurface is designed to support the propagation of a surface wave with the same wavenumber. Finally, the third metasurface is again designed as a phase-gradient but this time with increasing phase in the −*x*-direction, which reduces the momentum of the surface wave and hence transforms it back into a space wave.

### Experimental Demonstration: The “Electromagnetic Periscope”

#### Realization

**Figure 4:**
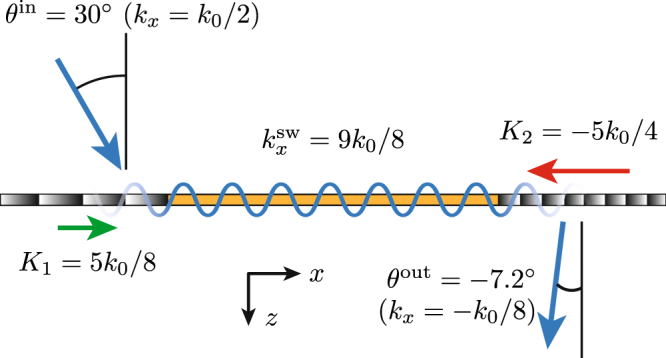
Schematic representation of the electromagnetic periscope metasurface system.

A schematic of the metasurface system is presented in Fig. 4. The figure shows the conceptual operation of the structure with momentum “push” (*K*_1_) and momentum “pull” (*K*_2_) induced by the first and last metasurfaces, respectively, and the surface-wave guidance in the middle metasurface. We shall now design the metasurface system for the following specifications: input angle *θ*^in^ = 30° and output angle *θ*^out^ = −7.2°.

The overall metasurface system, composed of the three juxtaposed metasurfaces, is implemented as a multilayer structure with three metallization layers and two dielectric spacers. The overall thickness of the structure is *λ*_0_/10. In each metasurface, the unit cell has a transverse size of *λ*_0_/5 × *λ*_0_/5 and includes in each layer a metallic scatterer in the form of a Jerusalem cross with specific geometric parameters^14–17^.

As said above, the *p*-polarization surface-wave guiding structure, in the middle of the metasurface system, is realized as a metasurface, for compatibility with its phase-gradient neighbours, instead of as a traditional waveguiding structure. In addition, to allow the *s*-polarization generalized refraction operation depicted in Fig. 1, this structure must be completely transparent, and could therefore not be implemented in the form of a conventional waveguide. The metasurface is designed so as to guide a *p*-polarized surface wave with an arbitrarily chosen propagation constant ${k}_{x}^{{\rm{sw}}}=9{k}_{0}/8$ at 10 GHz. In the current design, we consider the particular case of *s*-polarization normal transmission, leading to global uniformity.

**Figure 5:**
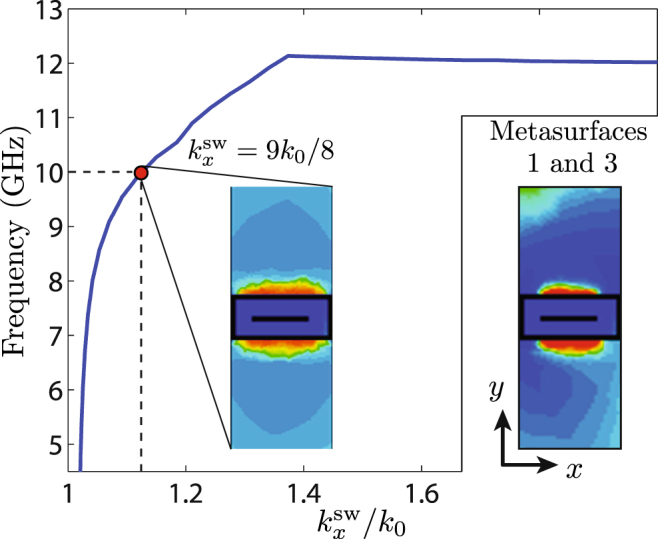
Dispersion curve and magnetic field distribution (absolute value at 10 GHz) for the fundamental mode of the waveguiding metasurface. The separate inset represents the excited fields in the surrounding phase-gradient metasurfaces (also at 10 GHz).

The waveguiding metasurface is uniform (as seen by a *p*-polarized wave), in contrast to the phase-gradient metasurfaces, only one unit cell has to be designed. Starting with an initial guess, the unit cell is optimized using an eigenmode solver with the goal to achieve a wavenumber of ${k}_{x}^{{\rm{sw}}}=9{k}_{0}/8$ at the operation frequency. The dispersion curve for the fundamental mode of the optimized waveguiding structure is plotted in Fig. 5. Note that the horizontal axis represents the *x*-wavenumber normalized to the free-space wavenumber, so that the figure shows only the slow-wave region (${k}_{x}^{{\rm{sw}}}/{k}_{0} > 1$).

The first phase-gradient metasurface, transforming the input *p*-polarized space wave into a surface wave, is implemented with a supercell of 8 unit cells of size *λ*_0_/5 with transmission phases ranging from 0 to 2*π*. The corresponding metasurface wavenumber is *K*_1_ = 2*π*/*P*_1_ = 2*π*/(8*λ*_0_/5) = 5*k*_0_/8. Then, for the specified input wave of *θ*^in^ = 30°, corresponding to ${k}_{x}^{{\rm{in}}}={k}_{0}/2$, one finds, using (1) with ${k}_{x}^{{\rm{i}}}={k}_{x}^{{\rm{in}}}$ and *K* = *K*_1_, the surface-wave wavenumber to be ${k}_{x}^{{\rm{sw}}}={k}_{x}^{{\rm{t}}}=9{k}_{0}/8$, which corresponds to the *x*-wavenumber across the entire metasurface system. The corresponding metasurface behavior is represented by the blue curve in Fig. 3, while the incidence angle (*θ*^i^ = 30°) is represented by the black star.

**Figure 6:**
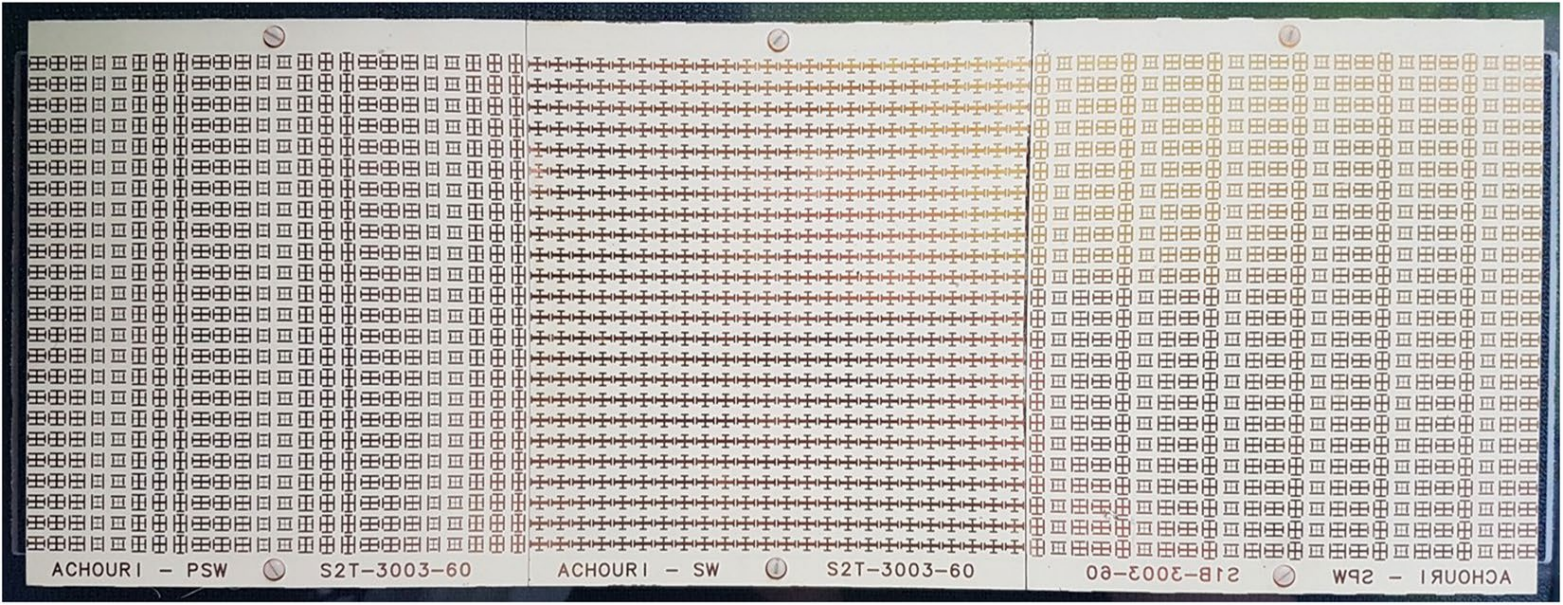
Fabricated metasurface system corresponding to Fig. 1. The metasurfaces from the left to the right perform the following operations on the *p*-polarized wave: space-wave to surface-wave transformation, surface-wave propagation, and surface-wave to space-wave transformation. At the same time, the central metasurface is perfectly transparent to *s*-polarized waves. The difference between the phase-gradients of the two end metasurfaces is clearly visible.

Upon this basis, the third metasurface is designed as follows. The output angle of *θ*^out^ = −7.2° corresponds to ${k}_{x}^{{\rm{out}}}=-{k}_{0}/8$. We apply (1) with ${k}_{x}^{{\rm{i}}}={k}_{x}^{{\rm{sw}}}=9{k}_{0}/8$ and ${k}_{x}^{{\rm{t}}}={k}_{x}^{{\rm{out}}}=-{k}_{0}/8$, which yields *K*_2_ = −5*k*_0_/4. Since |*K*_2_/*K*_1_| = 2, *P*_2_ = *P*_1_/2, and hence, still assuming *λ*_0_/5 unit cells, the supercell includes now 4 unit cells. The corresponding metasurface may be conveniently realized by cascading every two row of the first metasurface (see Fig. 6).

The exact dimensions of the Jerusalem crosses are found by numerical simulations using a commercial software. Each unit cell is simulated individually assuming periodic boundary conditions as approximate boundaries for smoothly varying patterns. The resulting scattering parameters, obtained from the simulations, are optimized by varying the dimensions of the crosses until the expected response is achieved^14–17^. For the two phase-gradient metasurfaces, the scattering parameters of each unit cell are assumed to simply consist of a phase transmission coefficient, *T* = *e*^*jϕ*^, where the phase shift *ϕ* depends on the unit cell position within the supercell.

Comparing the two insets in the figure shows that the field distribution of this fundamental mode of the waveguiding metasurface is essentially identical, and hence compatible, with the field distributions of the two phase-gradient metasurfaces. Since the metasurfaces have been in addition designed to all exhibit the same polarization and wavenumber, it may be inferred that the coupling between them is maximized, as desired.

The realized metasurface system is shown in Fig. 6. Due to limitation of our fabrication process, the three metasurfaces have been realized separately rather than as a single entity and have then been screwed to a plastic frame (at the back and hence not visible in Fig. 6) to form the overall metasurface system. Each metasurface is made of 24 × 24 unit cells, corresponding to a size of 4.8*λ*_0_ × 4.8*λ*_0_. The dimensions of the system are 45 cm × 15 cm × 3 mm.

#### Experiment

**Figure 7:**
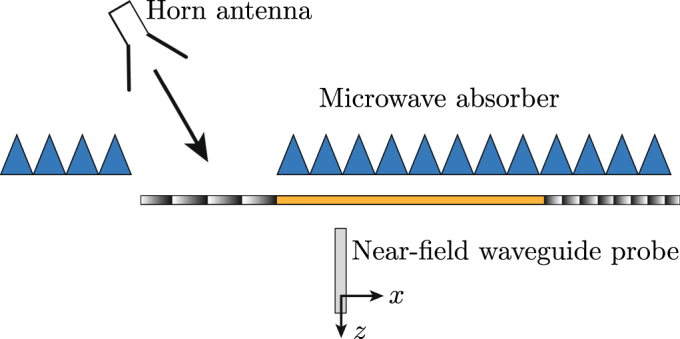
Side view of the metasurface system measurement setup.

The measurement of the metasurface system was performed using the experimental setup depicted in Fig. 7. The input side of the metasurface system is covered everywhere by absorbers except for a small aperture allowing the illumination of the first metasurface on the left. A high-gain X-band horn antenna illuminates the structure at the input side while a waveguide probe scans the metasurface system at the output side in the near-field region. Probe corrections are used to account for the directivity of the probe^18^. The near-field is measured in the middle of the metasurface system in Fig. 6 along the *x*-direction and at a distance of 0.7 cm from the metasurface. The measured near-field will be first Fourier-transformed to compute the spatial (*k*-domain) spectrum, and hence identify the modes excited at the output side of the system, and next propagated in the *xz*-plane by the angular spectrum technique^19^, so as to verify the periscope operation of the system.

**Figure 8:**
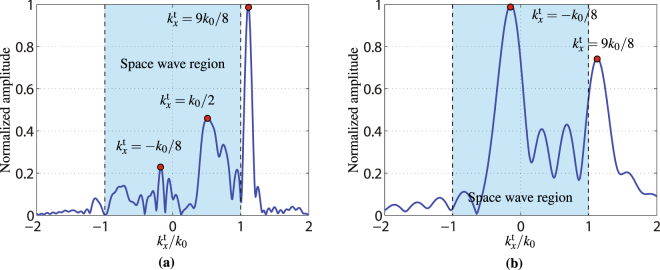
Normalized *x*-Fourier transform (*k*_*x*_-domain) of the output near-field measured along the *x*-direction at 1 cm from the metasurface in the *z*-direction (Fig. 7). (**a**) Scanning across the entire metasurface system. (**b**) Scanning only across the third metasurface. The regions highlighted in blue correspond to the radiation region.

The modes excited along the overall structure, as the probe scans the entire *x*- dimension of the system, are revealed in Fig. 8a, which plots the normalized *x*-Fourier transform of the output near-field measured along the *x*-direction using the setup of Fig. 7. The mode excited at the output of the metasurface system with the highest amplitude is a surface wave of wavenumber ${k}_{x}^{{\rm{sw}}}=9{k}_{0}/8$ corresponding to the wavenumber of the specified surface-wave mode. The reason why this mode is dominant is because it is excited along the entire structure, being first generated on the first metasurface, next guided by the second one and eventually radiated by the third one. The mode excited with the next higher intensity is the space-wave mode at ${k}_{x}^{{\rm{t}}}={k}_{0}/2$, which corresponds to the input wave impinging the metasurface at *θ*^in^ = 30°, which is present due to undesired scattering on the top and bottom sides of the metasurface system which could not be completely covered by the absorbers. The third largest peak lies in the negative side of the horizontal axis and corresponds to the specified transmitted space wave with wavenumber ${k}_{x}^{{\rm{t}}}=-\,{k}_{0}/8$ generated by the third metasurface.

Figure 8b shows the modes excited only at the output side of the third metasurface, when the near-field probe scans only on that part of the system. As expected, the two strongest modes correspond to the specified transmitted space wave with ${k}_{x}^{{\rm{t}}}=-\,{k}_{0}/8$ and the specified surface wave with ${k}_{x}^{{\rm{t}}}=9{k}_{0}/8$.

**Figure 9:**
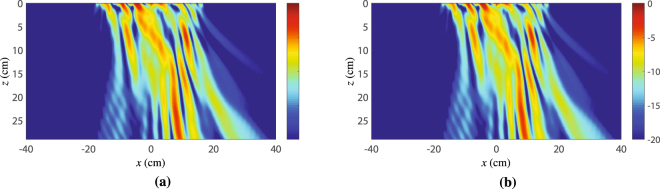
Absolute value of the transmitted electric fields (*E*_*x*_ component) obtained by angular spectrum propagation. (**a**) Without alteration of its angular spectrum. (**b**) After removing the incident wave contribution [range $0.2 < {k}_{x}^{{\rm{t}}}/{k}_{0} < 0.8$ in Fig. 8a]. The metasurface system is at *z* = 0 and extends from *x* = −22.5 cm to *x* = 22.5 cm.

Next, we compute the field scattered from the metasurface system by applying the angular spectrum propagation technique^19^ to the near-field measured along the entire structure. The resulting propagated scattered field is shown in Fig. 9a, where the metasurface system lies at *z* = 0 and extends from *x* = −22.5 cm to *x* = 22.5 cm. To clearly see the propagation of the expected transmitted space wave with ${k}_{x}^{{\rm{t}}}=-\,{k}_{0}/8$, we ignore the contribution of the input wave, which generates important spurious scattering, as is visible in Fig. 8a around ${k}_{x}^{{\rm{t}}}/{k}_{0}=0.5$. This is achieved by first taking the Fourier transform of the near-field, yielding the data in Fig. 8a, and next setting to zero all the modes excited in the region $0.2 < {k}_{x}^{{\rm{t}}}/{k}_{0} < 0.8$ in Fig. 8a to remove the contributions from the input wave. Then, the field is propagated along the *z*-direction following the usual procedure of the angular spectrum propagation technique. The resulting scattered field is plotted in Fig. 9b. In this figure, we can see the presence of a strong surface wave near the structure close to *z* = 0. In the region around *x* = −10 cm, we see some scattering which is due to the discontinuity between adjacent metasurfaces. In the region around *x* = 10 cm, we see a beam emerging from the metasurface system and being deflected towards the left. This beam corresponds to the specified transmitted space wave with *θ*^out^ = −7.2°.

**Figure 10:**
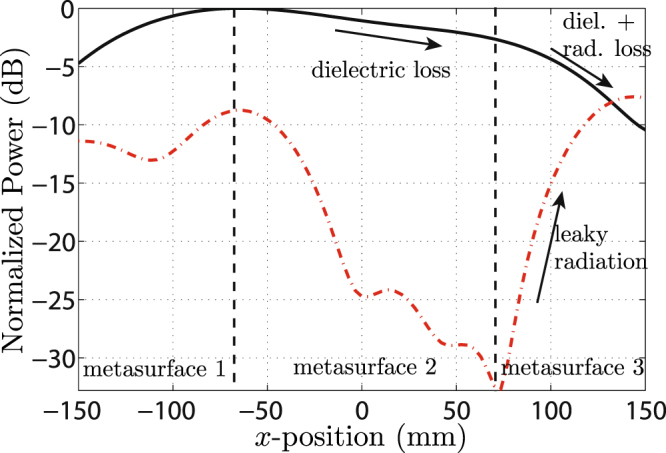
Normalized power distribution of the surface-wave mode (solid black line) and of the transmitted wave (dashed red line) over the metasurface system. The two vertical dashed black lines indicate the separation between the three metasurfaces.

In order to better understand the result shown in Fig. 9, we next analyze the spatial power distributions of the surface wave (${k}_{x}^{{\rm{sw}}}=9{k}_{0}/8$) and of the transmitted space wave (${k}_{x}^{{\rm{out}}}=-\,{k}_{0}/8$) along the metasurface system. From the data plotted in Fig. 8, it is possible to extract the power distribution of the different modes over the metasurface system. This is achieved by first isolating the modes of interest in the data of Fig. 8a by setting to zero everything except the relevant regions (appropriate peaks)–for example leaving only the peak centered at ${k}_{x}^{{\rm{t}}}=9{k}_{0}/8$ to isolate the surface wave–and then taking the inverse Fourier transform to generate the spatial distribution of the mode. The results are presented in Fig. 10, where the power distribution of the surface wave is represented by the solid black line and that of the space wave by the dashed red line.

As one moves along the *x*-axis, the power distribution of the surface wave (solid black curve) first increases, following the power distribution of the exciting horn antenna, which points at the junction between the first and second metasurfaces. At this point, it reaches a corresponding maximum. Then, it decreases as the wave propagates along the waveguiding metasurface while experiencing metallic and dielectric dissipation losses. Finally, it further decreases on the third metasurface due to combined dissipation and radiation losses.

The power level of the transmitted space wave (dash red curve) is relatively high at the junction between the first and second metasurfaces, which is explained by spurious scattering of the incident wave at this discontinuity. Then, this power rapidly decreases along the second metasurface, as expected from the fact that this surface does not radiate. Along the third metasurface, the power of the space wave progressively increases as it is progressively generated in terms of leaky-wave radiation by the interaction between the surface wave and the phase-gradient of the metasurface.

The experimental results presented above are in perfect agreement with the expected response of the metasurface system, with the exception of a relatively low radiation efficiency of about 10%. This low efficiency is due to a combination of effects that include surface-wave dissipation loss, scattering at each of the two metasurface discontinuities, the limited coupling of the incident wave which is effectively converted to a surface wave, and the imperfect conversion between space wave and surface wave (and vice-versa). Several of these issues may be addressed by further optimization. For instance, the discontinuity between the metasurfaces could be made more gradual, so as to avoid the generation of undesired scattering. Alternatively, the discontinuity may be smoothed out and coupling may be realized by a coupling metasurface placed below the structure, that would play the same role as the coupling waveguide in Fig. 2a. In such a strategy, the overall structure would have the exactly same configuration as that in Fig. 2a with the prisms replaced by the first and third metasurfaces.

### Other Potential Applications

The concept of space-wave via surface-wave routing may lead to a diversity of other potential applications. As an illustration, we will discuss two of them in this section.

#### Compact Beam Expander

An optical beam expander is a device that is used in telescopes or microscopes: it increases (or decreases) the lateral size of the incoming beam. The simplest way to realize such a device is to cascade two thin lenses of different focal lengths. We propose here an alternative beam expanding system, based on the concept of space-wave via surface-wave routing. Compared to the lens system, this routing system presents two significant advantages. First, it uses a single (composite) metasurface instead of two lenses. Second, in contrast to the lens system, it does not require any separation distance, where such a distance at optical frequencies represents several thousands of wavelengths, and hence leads to a very compact system.

**Figure 11:**
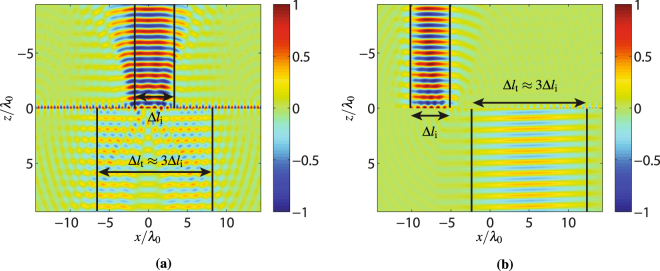
FDFD simulation of a beam expander with (**a**) direct transformation and (**b**) offset transformation. The metasurface system is designed to increase the beamwidth of the incident wave by a factor 3.

We present here two different beam expander designs, both increasing the beam width by a factor 3. One performs a direct conversion (without any lateral shift) while the other one performs an offset (laterally shifted) beam expansion. The direct beam expander is made of three metasurfaces, the middle one transforming the incident beam into two contra-propagating surface waves that are then both transformed back into space waves by the two end metasurfaces. This system is mathematically synthesized using the metasurface susceptibility synthesis technique developed in^6^. Once the metasurface susceptibilities are obtained, they are numerically simulated using the finite-difference frequency-domain technique presented in^20^. The resulting simulation showing this direct expansion is presented in Fig. 11a. As may be seen in this plot, the presence of the two metasurface discontinuities induces important spurious scattering.

The simulation of the offset beam expander is shown in Fig. 11b. The system is identical to that of Fig. 4 except that both the incident and transmitted angles are now normal to the surface. For the two structures in Fig. 11, the beam expansion of the transmitted wave is about three times that of the incident wave. Consequently, the amplitude of the transmitted wave is also three times less.

#### Multi-wave Refractor

**Figure 12:**
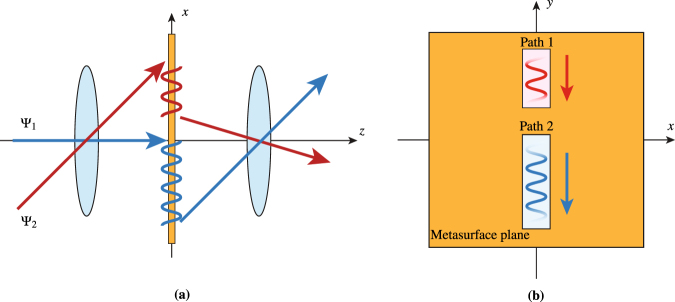
Multi-wave refractor system. (**a**) A 4 -*f* system, with 2 routing metasurface systems in its Fourier plane, which refracts the input waves Ψ_1_ and Ψ_2_ into different directions. (**b**) Corresponding representation of the metasurface Fourier plane with the two optical routes shifting the waves Ψ_1_ and Ψ_2_ to different locations in the Fourier plane.

The capability to route beams via surface waves may also be leveraged to implement a multi-wave refractor, i.e. system performing several refractive transformations with a single metasurface system, in contrast to a conventional metasurface that can only perform two independent refraction transformations, one for an *x*-polarized wave and one for a *y*-polarized wave (or up to 4 refractions by leveraging nonreciprocity and making use of gain and loss, as discussed in^21^). The proposed system is realized by inserting a metasurface at the Fourier plane of an optical 4-*f* system. A 4-*f* system is generally used as a spatial filter where a mask is placed at the Fourier plane to filter out certain spatial components of the incident wave^22^. Here, the metasurface placed at the Fourier plane is not used to filter out spatial components but, instead, to shift the spatial components of the incident waves to another region of the plane, which effectively changes the direction of propagation of the transmitted waves. The concept is depicted in Fig. 12a, where two input beams, Ψ_1_ and Ψ_2_, are transformed in terms of their spectral contents in the 4-*f* system.

The first lens focalizes the two beams at different locations in the Fourier plane, where a metasurface system is placed. This metasurface system consists of two “optical routes”, as shown in Fig. 12b, each composed of three different metasurfaces successively transforming the incident space wave into a surface wave, guiding this surface wave along the Fourier plane to the appropriate (*k*_*x*_, *k*_*y*_) point, and transforming it back into a space wave in the desired direction. The implementation of these “optical routes” would be based exactly on the same principle of the “periscope” discussed previously. In this example, the two beams have been shifted along the −*x*-direction in the Fourier plane. Their respective momenta along *x* have therefore been decreased. Consequently, the two beams exit the system, collimated by the second lens, with transmission angle depending on the points to which they have been shifted in the Fourier plane. Such a metasurface system might be populated with several additional “optical routes” so as to achieve even more refraction transformations.

## Discussion

In this work, we have introduced the concept of space-wave via surface-wave routing system composed of several juxtaposed metasurfaces. We have used a synthesis approach that consists in using phase-gradient metasurfaces, to generate the surface wave, and dispersion engineering, to guide the surface wave along the structure. This synthesis technique is based on the generalized law of refraction, which is approximate, and therefore leads to a less efficient structure, due to undesired scattering, but offers the advantage of being easier to realize.

As a proof of concept, we have presented a metasurface system acting as an “electromagnetic periscope”. This system spatially shifts an incident beam impinging the structure under a given angle and then reradiates it under a small angle. This structure produces the expected result, but suffers from a relatively low efficiency, which may be improved by further optimization. To illustrate the capabilities of the proposed concept, we have also presented two other potential applications, namely a compact beam expander and a multi-wave refractor that may be used as a spatial coupler between multiple inputs and outputs.
