# Author Correction: Space-Wave Routing via Surface Waves Using a Metasurface System

**DOI:** 10.1038/s41598-018-28343-8

**Published:** 2018-07-03

**Authors:** Karim Achouri, Christophe Caloz

**Affiliations:** Ecole polytechnique de Montreal, Department of Electrical Engineering, Montreal, QC H3T 1J4 Canada

Correction to: *Scientific Reports* 10.1038/s41598-018-25967-8, published online 15 May 2018

This Article contains an error in Figure 9, where Figure 9b is a duplication of Figure 9a. The correct Figure 9 appears below as Figure 1.

**Figure 1:**
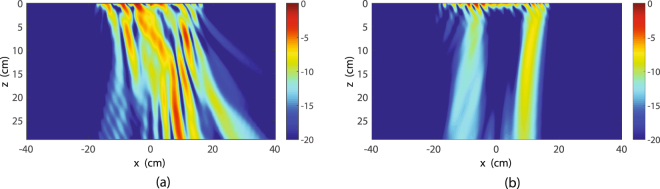
Absolute value of the transmitted electric fields (Ex component) obtained by angular spectrum propagation. (**a**) Without alteration of its angular spectrum. (**b**) After removing the incident wave contribution [${\rm{range}}\,0.2 < {k}_{x}^{{\rm{t}}}/{k}_{0} < 0.8$ in Fig. 8a]. The metasurface system is at *z* = 0 and extends from *x* = −22.5 cm to *x* = 22.5 cm.

